# Supplementary material for: Global transcriptional analysis of Geobacter sulfurreducens gsu1771 mutant biofilm grown on two different support structures
Source: PLoS One. 2023 Oct 25;18(10):e0293359. doi: 10.1371/journal.pone.0293359 (PMC10599522; doi:10.1371/journal.pone.0293359)
Supplement: S1 Fig — SDS-PAGE of protein used as a loading control in heme-staining (A) and western blot for OmcS (B), OmcZ (C) and PilA (D). The PageRuler Pre-stained Protein Ladder standard (ThermoScientific) was used as a molecular weight. (DOCX) [file pone.0293359.s001.docx]

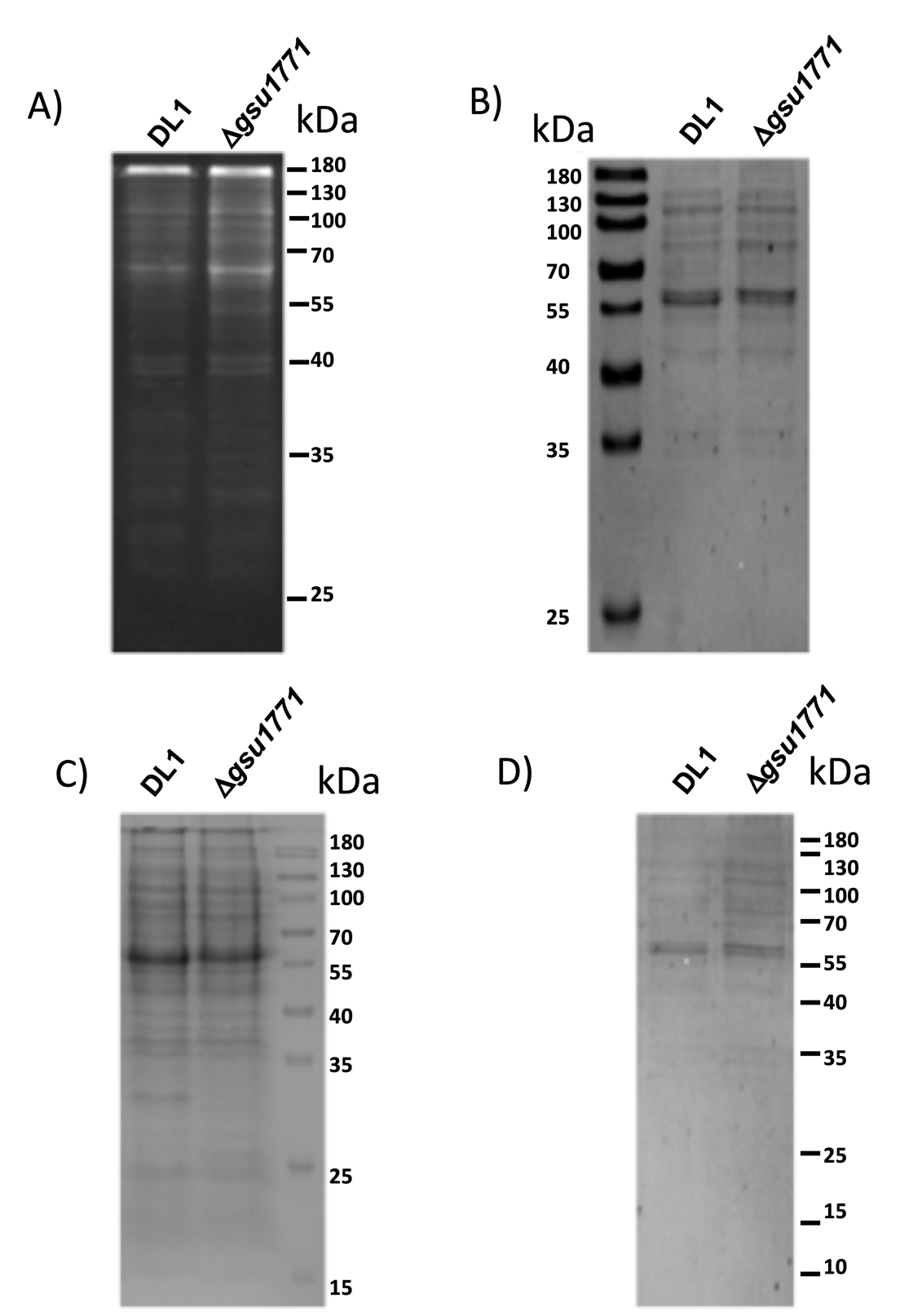


**S1 Fig.** SDS-PAGE of protein used as a loading control in heme-staining (A) and western blot for OmcS (B), OmcZ (C) and PilA (D). The PageRuler Pre-stained Protein Ladder standard (ThermoScientific) was used as a molecular weight.
