# Supplementary material for: Global transcriptional analysis of Geobacter sulfurreducens gsu1771 mutant biofilm grown on two different support structures
Source: PLoS One. 2023 Oct 25;18(10):e0293359. doi: 10.1371/journal.pone.0293359 (PMC10599522; doi:10.1371/journal.pone.0293359)
Supplement: S1 Table — (DOCX) [file pone.0293359.s002.docx]

**S1 Table. Bacteria, plasmid, and oligonucleotides used in this study.**

| **Name** | **Description** | **Reference** |
| --- | --- | --- |
| **Strains/Plasmids** | |  |
| *Geobacter sulfurreducens* |  |  |
| DL1 | Wild type | 1 |
| ∆*gsu1771* | DL1 with *gsu1771* deletion | 2 |
| *Escherichia coli* |  |  |
| MC1061 | Δ(*araA-leu*)7697 Δ(*lac*)X74 *galK* *galU* *hsr* *rpsL* *araD*139 | 3 |
| pBAD/His-GSU1771 | Plasmid for GSU1771 expression | 2 |
|  |  |  |
| **Oligonucleotides for RT-qPCR** | |  |
| qPCRepsHfw | CTATGCAGCCCTCACCATCC | This work |
| qPCRepsHrv | GTGAATGACATTGCCCTCGC | This work |
| qPCRomcMfw | TGGAGACTACCCATGCTGAA | 4 |
| qPCRomcMrev | AGACGTCGAGGTGCTCGTAT | 4 |
| qPCRaroG2fw | CAATGCCCAGAACTTTGCCC | This work |
| qPCRaroG2rv | TCATGACATACTCGGCGCTC | This work |
| qPCRhybAfw | GTGGCCTACAACAAGAACGC | This work |
| qPCRhybArv | ATCGCACTTGACGATCTGGG | This work |
| qPCRpgcAFw | GTCTCCAGAGTGCCGTAAGC | 2 |
| qPCRpgcARev | AGTAGGAGCCACTGCCAAGA | 2 |
| qPCR0941fw | GGTCCTTTCCTTTACCCGCC | This work |
| qPCR0941rv | GAGCGTTTATGGGCCTCGTA | This work |
| qPCRpanCfw | CTATCTCCACGACGGACACG | This work |
| qPCRpanCrv | TGGTGAAATCCTCGTTGGGG | This work |
| qPCR0972fw | AACTCACGTCGCTCATGGAC | This work |
| qPCR0972rv | ATGAACACGTCGGAGGGATG | This work |
| qPCR2507fw | TCGGCCGTGACTATTCGTTC | This work |
| qPCR2507rv | GCGGTGAACATTACAACGGG | This work |
| qPCRpilAfw | TCGGTATTCTCGCTGCAAT | 5 |
| qPCRpilArev | AATGCGGACTCAAGAGCAGT | 5 |
| qPCRdcuBFw | GCCATCAAGACCGGTATCC | 5 |
| qpCRdcuBrev | AGCCGAGGAAAAAGGTCACT | 5 |
| qPCRacnAFw | CCCGCTACAACTACCACTCC | 5 |
| qPCRacnARev | CGCAAAAGGTTTTCCAGAAG | 5 |
| qPCR0810fw | TGAGGGACACACCGACAATG | This work |
| qPCR0810rv | TCCCGAAATTGTCCACCAGG | This work |
| qPCRcsrAfw | CATCGAGGCTCCCAACGAC | This work |
| qPCRcsrArv | GTTACCGCCTTCTCCAGGTC | This work |
| qPCRpurE2fw | GGCCCATAAGGAAGGGGTG | This work |
| qPCRpurE2rv | AACCGATGACCGGCAATCTC | This work |
| qPCRato1fw | CATCGCCAACGCTGTAATCG | This work |
| qPCRato1rv | ATCGAACAGGTCGAGCATGG | This work |
| qPCR3356fw | GCGCTTTCTTCATCGCTATC | 4 |
| qPCR3356rv | GCAACCCCTGATTAGTCTCG | 4 |
| qPCRppcDfw | CAGCACTCACCCTGTTCTGT | 4 |
| qPCRppcDrev | TGCTTTTTGTGGTCGAAGGT | 4 |
|  |  |  |
| **Oligonucleotides for EMSA** | |  |
| pgcAemsaFw | CGCGCAAAACAATTCAGAC | 5 |
| pgcAemsaRv | CCTGAGCGCGTGGTAGTGT | 5 |
| gsu1771emsaFw | CGCTGGATCTCCTGAAAGG | 5 |
| gsu1771emsaRv | CGCGATACACCCTTACCTT | 5 |
| pulFemsaFw | ACCATCTTCGCATCCCTGTG | This work |
| pulFemsaRv | CTCCGAGGAGCCGAGCTTAC | This work |
| gsu3356emsaFw | ATCCATGCACTCCATCCTCC | This work |
| gsu3356emsaRv | AGGCTTGCTCGTCTCATGTC | This work |
| omcMemsaFw | CTCCAGCGTAATCAACGGCG | This work |
| omcMemsaRv | GGCTATCCTCTCTTTGCGTG | This work |
| omcBCemsaFw | AATTCGTCCGGATGTTTGTC | 2 |
| omcBCemsaRv | AAGTGGACCTCCTTTCTTCATTC | 2 |
| IG_303F | GTGGTATTCATGCACGTATTCTAATG | 2 |
| IG_303R | GAAGGGAAAGCTCATCCTATCC | 2 |

**References**

1. Caccavo FJr, Lonergan DJ, Lovley DR, Davis M, Stolz JF, Mclnerney MJ. *Geobacter sulfurreducens* sp. Vo., a hydrogen- and acetate-oxidizing dissimilatory metal-reducing microorganism. Appl Environ Microbiol. 1994, 60(10):3752-3759. doi:10.1128/aem.60.10.3752-3759.1994.

2. Hernández-Eligio A, Huerta-Miranda GA, Martínez-Bahena S, Castrejón-López B, Miranda-Hernández M, Juárez K. GSU1771 regulates extracellular electron transfer and electroactive biofilm formation in *Geobacter sulfurreducens*: Genetic and electrochemical characterization. Bioelectrochem. 2022, 145:108101. doi:10.1016/j.bioelechem.2022.108101.

3. Casadaban MJ, Cohen SN. Analysis of gene control signals by DNA fusion and cloning in Escherichia coli. J Mol Biol. 1980, 138(2):179-207. doi: 0.1016/0022-2836(80)90283-1.

4. Hernández-Eligio A, Pat-Espadas A, Vega-Alvarado L, Huerta-Amparan M, Cervantes FJ, Juárez K. Global transcriptional analysis of *Geobacter sulfurreducens* under palladium reducing conditions reveals new key cytochromes involved. Appl Microbiol Biotechnol. 2020, 104(9):4059–4069. doi:10.1007/s00253-020-10502-5.

5. Andrade A, Hernández-Eligio A, Tirado AL, Vega-Alvarado L, Olvera M, Morett E, Juárez K. Specialization of the reiterated copies of the heterodimeric integration host factor genes in *Geobacter sulfurreducens*. Front Microbiol. 2021, 12:626443. doi:10.3389/fmicb.2021.626443.
