# Supplementary material for: Global transcriptional analysis of Geobacter sulfurreducens gsu1771 mutant biofilm grown on two different support structures
Source: PLoS One. 2023 Oct 25;18(10):e0293359. doi: 10.1371/journal.pone.0293359 (PMC10599522; doi:10.1371/journal.pone.0293359)
Supplement: S2 Table — (DOCX) [file pone.0293359.s003.docx]

**S2 Table. List of differentially expressed genes in Δ*gsu1771* compared with the DL1 strain during biofilm formation on glass.**

| **Locus Tag** | **Name** | **Log2FC** | **pValue** | **Regulation** |
| --- | --- | --- | --- | --- |
| **Protein synthesis** | |  |  |  |
| GSU1709 | *smpB* | 1.6913194 | 7.594E-09 | UP |
| GSU1864 | *rsmA* | 1.9602557 | 1.068E-06 | UP |
| GSUR0060 | *rnpB* | -2.0156533 | 4.063E-26 | DOWN |
| GSUR031 | *ssrA* | -1.6842436 | 6.705E-17 | DOWN |
| GSU2686 | *rbbA* | -2.0013888 | 6.195E-16 | DOWN |
| GSU3235 | *rpmA* | -1.561657 | 6.429E-12 | DOWN |
| GSU1009 | | -1.5848468 | 1.966E-08 | DOWN |
| **Carbohydrate metabolism** | | |  |  |
| GSU1980 | | 2.2773529 | 4.713E-10 | UP |
| GSU1973 | | 2.5041665 | 5.688E-09 | UP |
| GSU1978 | *epsI* | 2.9682019 | 1.932E-08 | UP |
| GSU1975 | | 2.8911481 | 1.988E-17 | UP |
| GSU1976 | | 2.3335085 | 6.145E-15 | UP |
| GSU1977 | | 2.4860608 | 3.201E-14 | UP |
| GSU1970 | *neuB* | 2.28764 | 5.463E-14 | UP |
| GSU1972 | | 2.6962605 | 1.118E-13 | UP |
| GSU1952 | | 1.653185 | 0.0002046 | UP |
| GSU1963 | | 2.5726142 | 3.861E-08 | UP |
| GSU1959 | | 2.4206052 | 8.293E-07 | UP |
| GSU1958 | | 3.0042133 | 1.738E-06 | UP |
| GSU1962 | | 2.5010731 | 1.972E-06 | UP |
| GSU1961 | | 2.6016857 | 7.715E-06 | UP |
| GSU1245 | | 1.6500377 | 8.362E-06 | UP |
| GSU1979 | *epsH* | 2.1162614 | 1.141E-05 | UP |
| GSU1892 | *kdsC* | 1.6246396 | 6.044E-05 | UP |
| GSU1621 | | 1.8199315 | 0.0001001 | UP |
| GSU1965 | | 1.613406 | 0.0001496 | UP |
| GSU2308 | *mleA* | -1.626162 | 0.0001615 | DOWN |
| GSU2302 | | -2.4761336 | 1.098E-34 | DOWN |
| GSU0803 | *gltA* | -2.2712738 | 3.303E-34 | DOWN |
| GSU1106 | | -1.8139803 | 3.858E-24 | DOWN |
| GSU0910 | | -1.8398038 | 8.978E-21 | DOWN |
| GSU0385 | | -2.0794133 | 1.646E-19 | DOWN |
| GSU0846 | *acnA* | -2.0192387 | 1.383E-17 | DOWN |
| GSU0193 | | -1.7299635 | 1.25E-13 | DOWN |
| GSU0626 | *gmd* | -1.8006496 | 7.354E-14 | DOWN |
| **Nucleotide metabolism** | | |  |  |
| GSU1895 | | 1.585704 | 1.063E-10 | UP |
| GSU1982 | | 1.5326816 | 1.202E-06 | UP |
| GSU0672 | | -3.1573823 | 1.914E-41 | DOWN |
| GSU0140 | | -2.0993034 | 1.561E-24 | DOWN |
| GSU2306 | | -2.9409681 | 2.463E-09 | DOWN |
| GSU1758 | | -1.5756895 | 2.695E-09 | DOWN |
| **DNA/RNA metabolism** | | |  |  |
| GSU1421 | *sbcD-1* | 2.3558442 | 1.228E-05 | UP |
| GSU0547 | *mutS-2* | -3.5038665 | 6.899E-78 | DOWN |
| GSU0446 | *rsmE* | -3.1580888 | 1.922E-55 | DOWN |
| GSU1448 | | -2.4791789 | 3.234E-32 | DOWN |
| GSU0997 | *mutM* | -2.1987059 | 4.665E-31 | DOWN |
| GSU1614 | | -2.4311634 | 3.361E-29 | DOWN |
| GSU0711 | | -1.9778928 | 5.938E-20 | DOWN |
| GSU1613 | | -1.7636037 | 1.792E-15 | DOWN |
| GSU2614 | *recJ* | -2.1663779 | 2.844E-15 | DOWN |
| GSU1725 | *sbC-2* | -1.9479083 | 1.128E-14 | DOWN |
| GSU1711 | | -2.0875956 | 1.309E-14 | DOWN |
| GSU3245 | | -1.7221159 | 5.106E-14 | DOWN |
| GSU0892 | | -1.778691 | 7.63E-12 | DOWN |
| GSU0035 | | -2.1740484 | 5.28E-10 | DOWN |
| **Aminoacid metabolism** | | |  |  |
| GSU0989 | | 2.5149645 | 3.432E-24 | UP |
| GSU1707 | | 2.5730163 | 2.363E-16 | UP |
| GSU3142 | *aroG-2* | 3.2277905 | 2.468E-32 | UP |
| GSU1183 | *metY-1* | 3.5829191 | 1.178E-45 | UP |
| GSU1953 | *asnB* | 1.979256 | 1.156E-07 | UP |
| GSU2074 | | 1.9809226 | 5.631E-06 | UP |
| GSU3408 | *ltaE* | 1.5176193 | 9.787E-05 | UP |
| GSU2487 | *cpkA* | -2.8250159 | 2.468E-40 | DOWN |
| GSU0918 | *metK-2* | -1.8903711 | 1.616E-24 | DOWN |
| GSU0377 | *gcvP1* | -1.5829907 | 1.767E-14 | DOWN |
| GSU1578 | | -2.5526141 | 4.231E-14 | DOWN |
| GSU2292 | *ald* | -1.7095211 | 6.673E-12 | DOWN |
| GSU0375 | *gcvT* | -1.5914138 | 2.367E-11 | DOWN |
| GSU2462 | *metX* | -1.5396908 | 4.607E-08 | DOWN |
| GSU1513 | | -2.4555212 | 3.028E-05 | DOWN |
|  | | **Proteolysis** |  |  |
| GSU1960 | *cysE* | 3.58107 | 1.228E-05 | UP |
| GSU2075 | *ozpA* | 2.1325501 | 3.154E-11 | UP |
| GSU0786 | *hybP* | 1.7202074 | 2.878E-16 | UP |
| GSU2675 | | -2.4649428 | 4.703E-42 | DOWN |
| GSU1079 | | -2.2564422 | 1.072E-19 | DOWN |
| GSU0969 | *ctpA-1* | -1.7358142 | 2.499E-18 | DOWN |
| GSU0080 | *degQ* | -1.6326446 | 9.392E-16 | DOWN |
| GSU1944 | | -3.3286321 | 1.585E-15 | DOWN |
| GSU1943 | | -2.8769333 | 1.621E-10 | DOWN |
| GSU0120 | *hyaP* | -2.3905867 | 2.068E-10 | DOWN |
| **Metabolism of cofactors and vitamins** | | | | |
| GSU1184 | *acpH* | 3.0995973 | 2.996E-16 | UP |
| GSU1706 | *panC* | 2.1348496 | 5.059E-08 | UP |
| GSU1708 | | 2.1576258 | 3.753E-07 | UP |
| GSU1705 | *panB* | 1.7458488 | 1.065E-05 | UP |
| GSU0807 | *citX* | 1.5747652 | 0.0007348 | UP |
| GSU1577 | *cobA* | -2.8509347 | 1.678E-25 | DOWN |
| GSU0203 | | -1.9611052 | 1.352E-22 | DOWN |
| GSU0215 | *folD-1* | -1.9882819 | 1.134E-17 | DOWN |
| **Lipid metabolism** | | |  |  |
| GSU2584 | | -4.7610548 | 7.07E-114 | DOWN |
| GSU0490 | *ato-1* | -2.4239358 | 4.788E-39 | DOWN |
| GSU0142 | *pgpA* | -1.9250492 | 1.84E-13 | DOWN |
| GSU0460 | *fab-1* | -1.5909506 | 1.297E-11 | DOWN |
| **Energy metabolism and electron transport** | | | | |
| GSU1717 | *cysD* | 5.3435723 | 4.001E-12 | UP |
| GSU0087 | *hdrE* | 2.8251763 | 1.773E-11 | UP |
| GSU0089 | *hdrG* | 2.7416622 | 8.762E-11 | UP |
| GSU0088 | *hdrD* | 2.090912 | 6.447E-10 | UP |
| GSU0085 | *hdrF* | 2.8703395 | 1.808E-13 | UP |
| GSU0091 | *hdrB* | 2.7262579 | 1.04E-14 | UP |
| GSU2937 | | 1.5816638 | 1.48E-14 | UP |
| GSU1761 | *pgcA* | 2.0293686 | 1.564E-16 | UP |
| GSU0092 | *hdrC* | 2.3842659 | 6.418E-09 | UP |
| GSU2808 | | 1.7281938 | 6.798E-09 | UP |
| GSU3615 | | 2.6252528 | 5.986E-08 | UP |
| GSU2495 | | 1.5716829 | 1.182E-06 | UP |
| GSU1442 | | 2.3551049 | 9.674E-06 | UP |
| GSU3265 | | 1.6065614 | 2.46E-05 | UP |
| GSU2294 | *omcM* | 5.3093368 | 0.002269 | UP |
| GSU0782 | *hybS* | 2.291224 | 3.188E-36 | UP |
| GSU0783 | *hybA* | 2.2565513 | 1.079E-32 | UP |
| GSU0785 | *hybL* | 2.2607874 | 7.065E-30 | UP |
| GSU0784 | *hybB* | 2.3613566 | 3.577E-29 | UP |
| GSU0090 | *hdrA* | 2.7489255 | 9.842E-26 | UP |
| GSU1640 | *cydA* | 2.2535376 | 4.623E-22 | UP |
| GSU1718 | *cysN* | 2.8185537 | 7.875E-18 | UP |
| GSU0593 | | -1.7174898 | 1.246E-12 | DOWN |
| GSU1497 | *pilA-C* | -2.0648276 | 2.465E-14 | DOWN |
| GSU0220 | *coxC* | -1.628055 | 2.052E-09 | DOWN |
| GSU0200 | | -3.1950402 | 6.922E-51 | DOWN |
| GSU0122 | *hyaL* | -3.2799881 | 1.353E-50 | DOWN |
| GSU2657 | *ompC* | -3.4809886 | 2.084E-49 | DOWN |
| GSU0067 | *can-1* | -3.0051957 | 4.243E-48 | DOWN |
| GSU0075 | | -2.7984228 | 2.147E-46 | DOWN |
| GSU0219 | *coxA* | -2.7232687 | 2.235E-45 | DOWN |
| GSU0466 | *macA* | -2.5002545 | 5.091E-40 | DOWN |
| GSU1024 | *ppcD* | -3.0954513 | 1.656E-38 | DOWN |
| GSU0218 | *sco* | -2.6224497 | 6.612E-38 | DOWN |
| GSU3304 | *ompJ* | -2.4496632 | 1.607E-33 | DOWN |
| GSU0123 | *hyaS* | -2.9098786 | 5.079E-32 | DOWN |
| GSU0202 | | -2.2910925 | 1.134E-30 | DOWN |
| GSU0068 | | -2.6385568 | 4.127E-29 | DOWN |
| GSU0746 | | -2.5031884 | 4.079E-27 | DOWN |
| GSU0069 | | -2.4318906 | 1.516E-26 | DOWN |
| GSU2063 | | -2.1777753 | 5.433E-26 | DOWN |
| GSU2811 | | -1.9704253 | 2.264E-25 | DOWN |
| GSU3424 | *lpdA-3* | -2.0477721 | 3.608E-23 | DOWN |
| GSU0070 | | -2.3600007 | 4.4E-22 | DOWN |
| GSU2743 | | -1.9312371 | 1.484E-21 | DOWN |
| GSU0223 | *ctaB* | -2.107417 | 8.138E-21 | DOWN |
| GSU0195 | | -1.9839005 | 1.639E-19 | DOWN |
| GSU1394 | *ompB* | -1.5402306 | 1.003E-17 | DOWN |
| GSU0217 | | -3.5105913 | 2.868E-86 | DOWN |
| GSU1877 | | -3.7549842 | 8.968E-86 | DOWN |
| GSU0201 | | -2.9563525 | 2.277E-60 | DOWN |
| GSU1740 | | -2.779001 | 1.802E-16 | DOWN |
| GSU0121 | *hyaB* | -2.6399314 | 2.915E-15 | DOWN |
| GSU3259 | | -1.6111074 | 4.407E-15 | DOWN |
| GSU1496 | *pilA-N* | -2.137287 | 4.819E-15 | DOWN |
| GSU2724 | | -1.6410836 | 6.563E-15 | DOWN |
|  | **Transport** |  |  |  |
| GSU1985 | | 1.8843347 | 6.558E-13 | UP |
| GSU1380 | *feoB-1* | 1.8440717 | 2.683E-16 | UP |
| GSU0855 | | 1.9940533 | 5.682E-11 | UP |
| GSU3271 | | 1.8428379 | 6.403E-11 | UP |
| GSU3268 | | 1.6616427 | 7.689E-11 | UP |
| GSU3269 | | 1.8025749 | 0.0082846 | UP |
| GSU0972 | | 2.1962797 | 1.356E-25 | UP |
| GSU3270 | | 1.8815394 | 1.087E-09 | UP |
| GSU2939 | | 1.8592708 | 1.46E-20 | UP |
| GSU2135 | | 2.3801002 | 1.159E-08 | UP |
| GSU1778 | *pulQ* | 1.5966157 | 2.627E-08 | UP |
| GSU0815 | | 1.5608299 | 5.059E-08 | UP |
| GSU2136 | | 3.7822441 | 1.98E-07 | UP |
| GSU1528 | | 1.8367664 | 2.875E-07 | UP |
| GSU1923 | *lptG* | 1.6384729 | 3.383E-06 | UP |
| GSU1279 | *nukMN* | 2.390643 | 4.011E-06 | UP |
| GSU1784 | *pulF* | 1.7639177 | 4.646E-06 | UP |
| GSU1262 | | 2.1647756 | 2.578E-05 | UP |
| GSU1346 | *cysP* | 3.0455964 | 2.753E-05 | UP |
| GSU1922 | *lptF* | 1.7404824 | 0.0001032 | UP |
| GSU1900 | | 1.5461854 | 0.0002681 | UP |
| GSU1777 | *pulG* | 2.1317516 | 0.0003993 | UP |
| GSU1779 | *pulP* | 2.2857652 | 0.0008281 | UP |
| GSU1774 | *ftsX* | 2.300533 | 0.0009912 | UP |
| GSU1338 | | -2.6443987 | 0.0019874 | DOWN |
| GSU2008 | | -1.6623477 | 0.0057899 | DOWN |
| GSU2491 | | -1.6785213 | 7.358E-10 | DOWN |
| GSU1557 | | -3.2748349 | 3.456E-71 | DOWN |
| GSU2005 | | -3.8385407 | 1.049E-40 | DOWN |
| GSU2751 | *dcuB* | -2.6063825 | 8.847E-36 | DOWN |
| GSU1331 | | -2.2753083 | 1.766E-32 | DOWN |
| GSU0769 | *rarD* | -2.8744467 | 6.811E-32 | DOWN |
| GSU2481 | *kdpB* | -2.2298702 | 2.849E-31 | DOWN |
| GSU1332 | | -2.0411273 | 4.483E-28 | DOWN |
| GSU1330 | | -2.1544921 | 1.186E-26 | DOWN |
| GSU0518 | *aplD* | -1.7773259 | 9.599E-09 | DOWN |
| GSU2480 | *kdpA* | -2.3965394 | 1.523E-25 | DOWN |
| GSU2823 | *ybhG* | -1.9653939 | 1.12E-23 | DOWN |
| GSU0188 | | -2.1889602 | 3.473E-07 | DOWN |
| GSU0882 | | -2.5843614 | 0.0002777 | DOWN |
| GSU2692 | | -2.0388138 | 4.641E-19 | DOWN |
| GSU2687 | | -2.0727577 | 8.433E-17 | DOWN |
| GSU2482 | *kdpC* | -2.0895436 | 2.603E-16 | DOWN |
| GSU1070 | *aslB* | -2.283774 | 6.079E-13 | DOWN |
| GSU2350 | | -1.7059973 | 5.23E-11 | DOWN |
| GSU1480 | | -1.5637388 | 2.215E-10 | DOWN |
| **Cell envelope** | |  |  |  |
| GSU0991 | | 2.948461 | 3.011E-23 | UP |
| GSU1010 | | 2.2352707 | 0.0001124 | UP |
| GSU1814 | *divlC* | 4.2425755 | 0.0018582 | UP |
| GSU0810 | | -2.9225218 | 1.616E-56 | DOWN |
| GSU0182 | | -3.3521506 | 1.058E-51 | DOWN |
| GSU2486 | | -2.3057569 | 2.217E-28 | DOWN |
| GSU2690 | | -2.8874855 | 5.413E-24 | DOWN |
| GSU0073 | | -1.7768032 | 2.373E-20 | DOWN |
| GSU0185 | | -1.8984981 | 5.884E-19 | DOWN |
| GSU0767 | | -1.7440342 | 6.7E-19 | DOWN |
| GSU0181 | | -1.6871743 | 5.605E-16 | DOWN |
| GSU1168 | | -1.9516943 | 1.561E-14 | DOWN |
| GSU2662 | | -2.6574374 | 7.879E-14 | DOWN |
| GSU2305 | | -2.9677046 | 8.64E-11 | DOWN |
| **Regulatory functions and transcription** | | | | |
| GSU1043 | | 1.5787704 | 3.958E-05 | UP |
| GSU0693 | | 1.5627525 | 0.0001862 | UP |
| GSU1443 | | 1.9932166 | 0.0004618 | UP |
| GSU2670 | | 1.9893202 | 0.0025792 | UP |
| GSU0399 | | 5.0499234 | 0.0043623 | UP |
| GSU0470 | | 2.4131104 | 2.274E-08 | UP |
| GSU0471 | | 2.5530968 | 1.401E-06 | UP |
| GSU1630 | | 1.6279663 | 2.983E-06 | UP |
| GSU2519 | *yjiM* | 1.65492 | 4.282E-06 | UP |
| GSU2980 | *nikR* | 1.5383681 | 5.017E-06 | UP |
| GSU1268 | | 3.4782722 | 5.263E-06 | UP |
| GSU2506 | | 1.8295322 | 1.678E-12 | UP |
| GSU1382 | *ideR* | 1.7908976 | 5.845E-12 | UP |
| GSU1265 | | 3.8781152 | 2.103E-10 | UP |
| GSU1379 | *fur* | 1.6823788 | 2.209E-09 | UP |
| GSU0534 | *iscR-1* | 2.4426273 | 1.88E-17 | UP |
| GSU1529 | | 3.7161669 | 7.713E-17 | UP |
| GSU2507 | | 2.0541402 | 2.53E-16 | UP |
| GSU2755 | | -3.1910908 | 2.302E-61 | DOWN |
| GSU2484 | *kdpE* | -3.2340922 | 2.159E-60 | DOWN |
| GSU1090 | | -2.7056684 | 6.094E-41 | DOWN |
| GSU1727 | *dnaK* | -2.6707732 | 2.459E-39 | DOWN |
| GSU3341 | *prkA* | -2.2029883 | 9.008E-30 | DOWN |
| GSU2753 | | -2.572806 | 5.224E-28 | DOWN |
| GSU2483 | *kdpD* | -2.1609877 | 5.491E-28 | DOWN |
| GSU2750 | | -2.0374359 | 4.631E-26 | DOWN |
| GSU0018 | | -2.0898256 | 1.384E-22 | DOWN |
| GSU0079 | | -1.801824 | 8.28E-21 | DOWN |
| GSU0266 | | -2.2810134 | 2.719E-11 | DOWN |
| GSU0596 | | -1.8186012 | 4.194E-11 | DOWN |
| GSU3363 | | -1.7573531 | 4.192E-10 | DOWN |
| GSU0700 | | -1.5530088 | 1.274E-15 | DOWN |
| GSU2964 | *modE* | -1.5560469 | 2.92E-15 | DOWN |
| GSU1342 | | -2.551113 | 4.708E-15 | DOWN |
| GSU0372 | | -1.6128144 | 1.288E-14 | DOWN |
| GSU3357 | | -1.751833 | 5.515E-14 | DOWN |
| GSU2581 | | -2.0938926 | 9.126E-14 | DOWN |
| GSU2044 | | -1.6936705 | 1.593E-13 | DOWN |
| GSU2987 | | -2.0014745 | 3.397E-09 | DOWN |
| GSU1303 | | -1.6570809 | 3.924E-09 | DOWN |
| GSU0681 | | -1.5571873 | 6.094E-09 | DOWN |
| GSU3041 | *csrA* | -3.6963192 | 2.709E-08 | DOWN |
| GSU1072 | | -1.8468609 | 2.5E-07 | DOWN |
| GSU0473 | | -1.5606061 | 2.612E-05 | DOWN |
| GSU0682 | | -1.5400832 | 4.966E-05 | DOWN |
| GSU0881 | | -2.5151816 | 0.0001358 | DOWN |
| GSU1299 | *cheW34H-2* | -2.3319418 | 0.0002164 | DOWN |
| GSU0879 | *cheV* | -3.5461969 | 0.0012906 | DOWN |
| **Signal transduction** | | |  |  |
| GSU1266 | *lepA* | 1.6907337 | 4.211E-06 | UP |
| GSU0895 | | 1.6780033 | 5.23E-11 | UP |
| GSU2236 | *relA* | 1.788535 | 1.047E-09 | UP |
| GSU2622 | | 2.8892607 | 3.926E-14 | UP |
| GSU1007 | | -2.9495829 | 1.076E-55 | DOWN |
| GSU0078 | | -2.2918323 | 1.55E-32 | DOWN |
| GSU1037 | | -2.9301663 | 5.182E-21 | DOWN |
| GSU1400 | | -2.4033929 | 3.828E-17 | DOWN |
| GSU3356 | | -2.0745551 | 5.541E-17 | DOWN |
| GSU1149 | | -1.6607129 | 1.86E-16 | DOWN |
| GSU1937 | | -1.798997 | 8.23E-14 | DOWN |
| GSU3033 | | -1.9223457 | 4.774E-07 | DOWN |
|  | **Others** |  |  |  |
| GSU1983 | | 1.6046259 | 3.377E-09 | UP |
| GSU3065 | *ftsQ* | 1.602163 | 5.21E-10 | UP |
| GSU1974 | | 2.3737296 | 2.661E-16 | UP |
| GSU2715 | | 1.9305365 | 2.61E-19 | UP |
| GSU2940 | | 1.6546097 | 6.741E-18 | UP |
| GSU1987 | | 1.8856454 | 1.289E-17 | UP |
| GSU2390 | *htpG* | 1.8919423 | 2.891E-17 | UP |
| GSU0033 | *dnaK* | 1.9667253 | 5.309E-25 | UP |
| GSU0993 | | 2.5607172 | 0.0011813 | UP |
| GSU0398 | | 3.0919804 | 0.0014659 | UP |
| GSU1387 | *cse4* | 1.6407957 | 0.0004891 | UP |
| GSU0052 | *csx14* | 1.6305906 | 0.0005088 | UP |
| GSU2235 | | 2.3630085 | 0.0001354 | UP |
| GSU2409 | | 1.5130147 | 1.634E-07 | UP |
| GSU2410 | | 1.5447808 | 2.828E-07 | UP |
| GSU2406 | | 1.6843921 | 5.688E-09 | UP |
| GSU2518 | | 1.910457 | 3.121E-07 | UP |
| GSU2888 | | 1.511843 | 9.163E-07 | UP |
| GSU1011 | | 1.707932 | 4.402E-06 | UP |
| GSU0685 | *hpnH* | 1.6531508 | 5.4E-06 | UP |
| GSU3055 | *flhF* | -3.1035874 | 3.429E-08 | DOWN |
| GSU3044 | *flgN* | -1.9394063 | 5.745E-08 | DOWN |
| GSU0847 | | -1.5919445 | 3.078E-07 | DOWN |
| GSU0414 | *fliJ* | -2.5951432 | 1.047E-06 | DOWN |
| GSU0417 | *flgD* | -3.1934089 | 2.981E-06 | DOWN |
| GSU0418 | | -4.2324402 | 7.036E-06 | DOWN |
| GSU2010 | | -1.5374651 | 3.132E-05 | DOWN |
| GSU0124 | | -1.6230182 | 3.607E-05 | DOWN |
| GSU2678 | | -2.2835093 | 4.569E-05 | DOWN |
| GSU3050 | *flgA* | -3.2424991 | 6.678E-05 | DOWN |
| GSU0427 | | -1.6470747 | 0.000102 | DOWN |
| GSU1551 | | -1.6682859 | 0.0001031 | DOWN |
| GSU3056 | *flhA* | -2.098079 | 0.0001609 | DOWN |
| GSU3015 | *flaG* | -1.7964258 | 0.0001685 | DOWN |
| GSU1344 | | -1.7499049 | 0.0010508 | DOWN |
| GSU3045 | *flgM* | -2.2946605 | 0.0011646 | DOWN |
| GSU1572 | | -1.9904313 | 0.0017936 | DOWN |
| GSU3054 | *flhG* | -2.8178023 | 0.004582 | DOWN |
| GSU0419 | *flgE* | -2.3475607 | 0.0091329 | DOWN |
| GSU1298 | | -1.7417385 | 0.0091329 | DOWN |
| GSU0194 | | -2.0882259 | 5.433E-26 | DOWN |
| GSU2471 | | -1.9494255 | 1.711E-23 | DOWN |
| GSU0955 | | -1.9189416 | 5.813E-23 | DOWN |
| GSU0416 | *fliK* | -3.8905823 | 4E-22 | DOWN |
| GSU3343 | | -1.8658974 | 4.908E-22 | DOWN |
| GSU3043 | *flgk* | -3.0135546 | 4.955E-22 | DOWN |
| GSU1307 | *ftn* | -2.0462964 | 4.968E-22 | DOWN |
| GSU2193 | | -2.2384265 | 1.225E-20 | DOWN |
| GSU0885 | | -1.9979934 | 1.646E-19 | DOWN |
| GSU0728 | *ppk-2* | -1.8854176 | 4.24E-17 | DOWN |
| GSU1404 | | -1.8506743 | 7.665E-17 | DOWN |
| GSU1830 | | -1.9025669 | 1.074E-16 | DOWN |
| GSU0544 | | -1.6276358 | 3.859E-16 | DOWN |
| GSU0768 | | -1.6276129 | 8.043E-16 | DOWN |
| GSU3152 | | -1.9490742 | 2.043E-15 | DOWN |
| GSU3040 | *fliW* | -4.0010345 | 2.277E-15 | DOWN |
| GSU0674 | *hcp* | -1.5491616 | 3.11E-15 | DOWN |
| GSU1087 | | -1.6962154 | 6.058E-14 | DOWN |
| GSU3329 | | -1.6800198 | 1.22E-12 | DOWN |
| GSU0196 | | -1.5577406 | 1.311E-12 | DOWN |
| GSU3349 | | -1.9018479 | 1.315E-12 | DOWN |
| GSU1642 | | -1.8734018 | 2.844E-12 | DOWN |
| GSU2788 | | -1.6677584 | 4.89E-12 | DOWN |
| GSU1370 | | -1.5388669 | 1.321E-10 | DOWN |
| GSU0517 | | -1.5457485 | 2.92E-10 | DOWN |
| GSU1670 | | -1.8079201 | 3.514E-10 | DOWN |
| GSU3289 | | -1.6037355 | 7.161E-10 | DOWN |
| GSU1560 | | -1.7222442 | 9.022E-10 | DOWN |
| GSU1579 | | -1.7725984 | 1.703E-09 | DOWN |
| GSU0931 | | -1.5368641 | 5.067E-09 | DOWN |
| GSU0548 | | -4.6734079 | 6.87E-121 | DOWN |
| GSU0066 | | -2.8999958 | 4.236E-51 | DOWN |
| GSU2536 | | -2.6184934 | 5.829E-46 | DOWN |
| GSU0352 | *prx-3* | -2.5545838 | 4.797E-40 | DOWN |
| GSU3042 | *flgL* | -4.517269 | 2.227E-36 | DOWN |
| GSU0893 | *prx-1* | -2.6255005 | 1.256E-33 | DOWN |
| GSU1728 | | -2.403064 | 7.171E-33 | DOWN |
| GSU0074 | *elbB* | -2.2130478 | 1.167E-31 | DOWN |
| GSU0720 | | -2.2050293 | 2.503E-31 | DOWN |
| GSU0804 | *wrbA* | -2.0986662 | 8.54E-29 | DOWN |
| GSU0802 | | -2.0528314 | 9.33E-28 | DOWN |
| **Unknown function** | | |  |  |
| GSU0467 | | 1.7334458 | 0.0018114 | UP |
| GSU3560 | | 6.2298032 | 0.000605 | UP |
| GSU0553 | | 1.7245327 | 0.0008283 | UP |
| GS_RS10695 | | 6.0023259 | 0.0014024 | UP |
| GSU0468 | | 1.8834272 | 4.031E-05 | UP |
| GSU1103 | | 1.8497813 | 0.0002183 | UP |
| GSU1846 | | 3.0247596 | 1.716E-05 | UP |
| GSU0590 | | 1.597198 | 1.115E-05 | UP |
| GSU1981 | | 1.912617 | 9.069E-06 | UP |
| GSU2756 | | 1.5093935 | 5.339E-08 | UP |
| GSU1971 | | 1.8293678 | 1.231E-08 | UP |
| GSU2936 | | 1.654133 | 8.595E-10 | UP |
| GSU2499 | | 1.8383918 | 2.488E-09 | UP |
| GSU0985 | | 2.2523854 | 1.542E-07 | UP |
| GSU3267 | | 1.7511736 | 2.14E-07 | UP |
| GSU0986 | | 2.0988145 | 5.166E-11 | UP |
| GSU1969 | | 1.8961545 | 6.092E-11 | UP |
| GSU3474 | | 2.8287719 | 7.8E-11 | UP |
| GSU0978 | | 2.5757744 | 9.34E-14 | UP |
| GSU0619 | | 2.2333461 | 6.021E-14 | UP |
| GSU0983 | | 2.3381266 | 7.849E-16 | UP |
| GSU0979 | | 2.1075849 | 5.689E-17 | UP |
| GSU3141 | | 2.6719639 | 1.875E-17 | UP |
| GSU0973 | | 2.5105273 | 2.337E-17 | UP |
| GSU0974 | | 2.127643 | 1.184E-17 | UP |
| GSU0980 | | 1.9641104 | 1.148E-19 | UP |
| GSU2505 | | 1.9471283 | 4.691E-20 | UP |
| GSU0976 | | 2.6071268 | 5.047E-22 | UP |
| GSU0982 | | 2.1521657 | 4.133E-21 | UP |
| GSU0977 | | 2.4414773 | 4.778E-21 | UP |
| GSU2496 | | 2.1488278 | 1.001E-22 | UP |
| GSU0988 | | 2.525925 | 1.623E-24 | UP |
| GSU0975 | | 2.538902 | 7.061E-32 | UP |
| GSU0990 | | 3.1814321 | 8.087E-32 | UP |
| GSU0987 | | 2.8136981 | 1.684E-35 | UP |
| GSU0992 | | 3.5096584 | 1.207E-34 | UP |
| GSU2585 | | -5.2442895 | 3.23E-113 | DOWN |
| GSU0071 | | -4.0164564 | 1.368E-90 | DOWN |
| GSU2586 | | -3.7170897 | 2E-61 | DOWN |
| GSU2583 | | -3.5219731 | 2.927E-60 | DOWN |
| GSU3351 | | -3.6353249 | 2.804E-57 | DOWN |
| GSU1684 | | -2.9033823 | 1.171E-55 | DOWN |
| GSU0472 | | -3.1397618 | 3.314E-52 | DOWN |
| GSU0444 | | -2.9641764 | 4.318E-44 | DOWN |
| GSU1171 | *yyaL* | -2.8244868 | 2.629E-42 | DOWN |
| GSU3409 | | -3.2607279 | 4.34E-42 | DOWN |
| GSU0968 | | -2.369761 | 1.361E-39 | DOWN |
| GSU0680 | | -2.3462881 | 4.254E-38 | DOWN |
| GSU3506 | | -3.8433517 | 7.543E-36 | DOWN |
| GSU0996 | | -2.6148133 | 1.311E-35 | DOWN |
| GSU0172 | | -2.7759131 | 3.303E-34 | DOWN |
| GSU3549 | | -2.4584824 | 2.503E-31 | DOWN |
| GSU2412 | | -2.5719965 | 2.924E-31 | DOWN |
| GSU3342 | | -2.3446976 | 3.728E-31 | DOWN |
| GSU0915 | | -2.9888684 | 4.412E-31 | DOWN |
| GSU0216 | | -2.5968427 | 1.449E-30 | DOWN |
| GSU3451 | | -2.4762571 | 3.54E-30 | DOWN |
| GS_RS07775 | | -2.7588973 | 8.214E-30 | DOWN |
| GSU2792 | | -2.028779 | 2.637E-27 | DOWN |
| GSU3425 | | -2.5788878 | 8.376E-27 | DOWN |
| GSU1212 | | -2.0602062 | 4.705E-26 | DOWN |
| GSU0710 | | -2.4197321 | 1.964E-25 | DOWN |
| GSU1726 | | -2.8322964 | 2.125E-25 | DOWN |
| GSU0709 | | -2.2864164 | 4.484E-25 | DOWN |
| GSU3473 | | -2.051711 | 2.061E-23 | DOWN |
| GSU0719 | | -1.9492099 | 4.397E-23 | DOWN |
| GSU3478 | | -2.9637275 | 4.209E-22 | DOWN |
| GSU1213 | | -2.1873379 | 4.968E-22 | DOWN |
| GSU1209 | | -1.9168487 | 1.186E-20 | DOWN |
| GSU0141 | | -1.7809665 | 8.839E-20 | DOWN |
| GSU3626 | | -2.2201109 | 2.012E-19 | DOWN |
| GSU3489 | | -1.9152818 | 1.451E-17 | DOWN |
| GSU0919 | | -1.6549919 | 1.486E-17 | DOWN |
| GSU0077 | | -2.0792469 | 2.418E-17 | DOWN |
| GSU2791 | | -1.8787733 | 2.405E-16 | DOWN |
| GSU0917 | | -2.0400838 | 6.096E-16 | DOWN |
| GSU2347 | | -2.0034935 | 9.54E-16 | DOWN |
| GSU1395 | | -1.5775369 | 1.614E-15 | DOWN |
| GSU0224 | | -2.1412003 | 1.752E-15 | DOWN |
| GSU1683 | | -1.9095924 | 2.58E-15 | DOWN |
| GSU3583 | | -1.5431345 | 7.694E-15 | DOWN |
| GSU3305 | | -1.6903418 | 2.755E-14 | DOWN |
| GSU2691 | | -2.7430621 | 3.923E-14 | DOWN |
| GSU3511 | | -1.6707805 | 6.418E-14 | DOWN |
| GSU0712 | | -1.912317 | 7.069E-14 | DOWN |
| GSU3151 | | -1.6936666 | 8.366E-14 | DOWN |
| GSU0173 | | -1.7703544 | 1.25E-13 | DOWN |
| GSU1995 | | -1.8153303 | 1.766E-13 | DOWN |
| GSU0717 | | -1.7427386 | 5.201E-13 | DOWN |
| GSU0081 | | -1.7605552 | 1.148E-12 | DOWN |
| GSU2747 | | -1.6855694 | 7.671E-12 | DOWN |
| GSU0597 | | -1.8712447 | 9.188E-11 | DOWN |
| GSU3410 | | -3.3755508 | 5.728E-10 | DOWN |
| GSU0584 | | -1.5468094 | 8.307E-10 | DOWN |
| GSU1615 | | -2.137929 | 2.512E-09 | DOWN |
| GSU0415 | | -2.5640667 | 7.726E-09 | DOWN |
| GSU1071 | | -2.32609 | 2.005E-08 | DOWN |
| GSU3014 | | -2.3799348 | 2.434E-08 | DOWN |
| GSU1669 | | -1.8454022 | 1.369E-07 | DOWN |
| GSU1308 | | -1.6731637 | 2.86E-07 | DOWN |
| GSU1447 | | -1.7206652 | 3.68E-07 | DOWN |
| GSU3452 | *slyX* | -1.6520453 | 1.048E-06 | DOWN |
| GSU1167 | | -2.6171251 | 2.805E-06 | DOWN |
| GSU2488 | | -1.6461946 | 2.962E-06 | DOWN |
| GSU2902 | | -1.7918231 | 3.808E-06 | DOWN |
| GSU1025 | | -2.9679069 | 4.645E-06 | DOWN |
| GSU2321 | | -1.7845979 | 7.938E-06 | DOWN |
| GSU3358 | | -1.5595742 | 1.086E-05 | DOWN |
| GSU3035 | | -2.398922 | 1.252E-05 | DOWN |
| GSU1339 | | -3.0956119 | 1.368E-05 | DOWN |
| GSU3034 | | -1.8874776 | 3.501E-05 | DOWN |
| GSU2348 | | -1.5431896 | 0.0002287 | DOWN |
| GSU3629 | | -2.5784392 | 0.000269 | DOWN |
| GSU3568 | *lnt-C* | -1.7708855 | 0.0002965 | DOWN |
| GSU0539 | | -2.1318523 | 0.0015446 | DOWN |
| GSU3589 | | -1.9902576 | 0.0019135 | DOWN |
