# Supplementary material for: Global transcriptional analysis of Geobacter sulfurreducens gsu1771 mutant biofilm grown on two different support structures
Source: PLoS One. 2023 Oct 25;18(10):e0293359. doi: 10.1371/journal.pone.0293359 (PMC10599522; doi:10.1371/journal.pone.0293359)
Supplement: S3 Table — (DOCX) [file pone.0293359.s004.docx]

**S3 Table.** **List of differentially expressed genes in Δ*gsu1771* compared with DL1 strain during biofilm formation on graphite electrode.**

| **Locus Tag** | **Name** | **log2FC** | **pValue** | **Regulation** |
| --- | --- | --- | --- | --- |
| **Amino acids metabolism** | | | |  |
| GSU1513 | | -2.9515969 | 1.55E-09 | DOWN |
| GSU2487 | *cpkA* | -1.5522397 | 4.80E-05 | DOWN |
| GSU2502 | | -1.9572199 | 1.02E-06 | DOWN |
| GSU0989 | | 2.61585862 | 2.01E-08 | UP |
| GSU3142 | *aroG-2* | 1.9413409 | 5.34E-06 | UP |
| **Carbohydrate metabolism** | | | |  |
| GSU1514 | | -3.1095079 | 1.88E-07 | DOWN |
| **Cell envelope** | |  |  |  |
| GSU0991 | | 3.19430338 | 2.43E-14 | UP |
| GSU3548 | *pilE* | 3.07715059 | 1.25E-08 | UP |
| **DNA/RNA metabolism** | | | |  |
| GSU0547 | *mutS-2* | -3.1919716 | 2.40E-10 | DOWN |
| GSU2614 | *recJ* | -2.4243314 | 1.45E-09 | DOWN |
| **Energy metabolism and electron transport** | | | | |
| GSU0592 | *omcQ* | 2.11835468 | 1.78E-07 | UP |
| GSU0702 | | 2.1291768 | 2.45E-07 | UP |
| GSU0782 | *hybS* | 3.42092329 | 5.52E-15 | UP |
| GSU1442 | | 3.98651371 | 1.98E-17 | UP |
| GSU1538 | | 4.03618048 | 7.67E-11 | UP |
| GSU1640 | *cydA* | 2.10734542 | 1.28E-06 | UP |
| GSU2294 | *omcM* | 3.97660242 | 2.06E-11 | UP |
| GSU2513 | | 1.91497648 | 4.43E-06 | UP |
| GSU2642 | | 2.6379398 | 9.90E-05 | UP |
| GSU2748 | | 2.16277266 | 2.23E-06 | UP |
| GSU2801 | | 2.34285405 | 2.32E-09 | UP |
| GSU2808 | | 2.6928883 | 9.51E-10 | UP |
| GSU2887 | | 1.66586 | 7.88E-05 | UP |
| GSU3615 | | 1.62833131 | 3.96E-05 | UP |
| GSU1238 | | -2.4421669 | 2.58E-07 | DOWN |
| GSU1394 | *ompB* | -1.9481029 | 2.60E-05 | DOWN |
| GSU1877 | | -3.3612947 | 9.32E-12 | DOWN |
| GSU2501 | | -1.6933054 | 4.55E-05 | DOWN |
| GSU2503 | *omcT* | -5.0280485 | 2.81E-28 | DOWN |
| GSU2504 | *omcS* | -4.9883293 | 4.71E-23 | DOWN |
| **Lipids metabolism** | | |  |  |
| GSU2584 | | -1.6103622 | 0.00137395 | DOWN |
| **Others** |  |  |  |  |
| GSU1154 | | 2.39158286 | 1.67E-07 | UP |
| GSU1556 | | 2.24039765 | 4.13E-07 | UP |
| GSU1905 | | 1.65016601 | 0.00052717 | UP |
| GSU1945 | | 2.09598262 | 0.00032591 | UP |
| GSU2410 | *hspA-2* | 3.33047798 | 9.71E-06 | UP |
| GSU2940 | | 1.94935111 | 0.000539 | UP |
| GSU2967 | | 4.03350281 | 2.67E-13 | UP |
| GSU0548 | | -3.4438109 | 6.84E-13 | DOWN |
| GSU1237 | | -2.0043179 | 6.61E-07 | DOWN |
| GSU2612 | | -1.8580094 | 3.56E-06 | DOWN |
| GSU3329 | | -1.8208294 | 6.11E-05 | DOWN |
| **Proteolysis** | |  |  |  |
| GSU0538 | *hspA-1* | 2.35707996 | 1.85E-05 | UP |
| GSU1079 | | -2.2397867 | 0.00171619 | DOWN |
| GSU1943 | | -2.7276643 | 3.97E-06 | DOWN |
| GSU1944 | | -2.6065485 | 0.00011875 | DOWN |
| **Regulatory functions and transcription** | | | | |
| GSU0470 | | 3.03271733 | 1.58E-11 | UP |
| GSU0471 | | 3.61223859 | 2.32E-12 | UP |
| GSU0596 | | 2.06077082 | 1.27E-05 | UP |
| GSU1264 | | 7.6044959 | 3.86E-22 | UP |
| GSU1265 | | 4.07773243 | 2.59E-17 | UP |
| GSU1268 | | 3.47914667 | 2.31E-13 | UP |
| GSU2670 | | 3.56252668 | 2.67E-13 | UP |
| GSU2749 | | 2.39680421 | 6.22E-07 | UP |
| GSU3261 | | 2.44994023 | 1.16E-07 | UP |
| GSU3370 | | 1.78348986 | 3.97E-06 | UP |
| GSU3419 | | 2.39599306 | 1.27E-07 | UP |
| GSUR0059 | *ssrS* | 3.02678209 | 6.21E-08 | UP |
| GSU1939 | | -2.2376667 | 2.23E-07 | DOWN |
| GSU2506 | | -2.2217854 | 5.20E-09 | DOWN |
| GSU2507 | | -1.5919082 | 9.74E-05 | DOWN |
| GSU2815 | | -2.4935195 | 2.53E-11 | DOWN |
| GSU3586 | | -1.6731693 | 6.91E-05 | DOWN |
| **Signal transduction** | | |  |  |
| GSU0537 | | 2.26011405 | 5.98E-05 | UP |
| GSU1037 | | -3.5653567 | 1.17E-11 | DOWN |
| **Transport** | |  |  |  |
| GSU0428 | *tssJ* | 7.88046637 | 6.19E-07 | UP |
| GSU0431 | *tssF* | 4.38121083 | 2.47E-06 | UP |
| GSU0433 | *tssH* | 2.41074911 | 7.96E-09 | UP |
| GSU0972 | | 2.44185511 | 8.37E-08 | UP |
| GSU1153 | | 2.03254851 | 4.43E-06 | UP |
| GSU2172 | | 4.36823165 | 6.35E-12 | UP |
| GSU2751 | *dcuB* | 1.92775634 | 0.00151464 | UP |
| GSU2939 | | 1.62027285 | 0.0044809 | UP |
| GSU3165 | *tssL* | 3.70517001 | 6.68E-07 | UP |
| GSU3166 | *tssM* | 3.87662774 | 5.52E-15 | UP |
| GSU3167 | *tssA* | 5.34924051 | 2.62E-15 | UP |
| GSU3174 | *tssD* | 4.12933706 | 2.50E-08 | UP |
| GSU2613 | *fieF* | -1.9215049 | 8.65E-06 | DOWN |
| **Unknown function** | | |  |  |
| GSU0597 | | 2.26899803 | 1.55E-05 | UP |
| GSU0619 | | 2.10806728 | 4.71E-08 | UP |
| GSU0973 | | 2.46675289 | 1.28E-09 | UP |
| GSU0974 | | 2.5656892 | 2.01E-08 | UP |
| GSU0975 | | 2.73149037 | 1.86E-07 | UP |
| GSU0976 | | 2.53278796 | 2.23E-07 | UP |
| GSU0977 | | 2.70582542 | 1.56E-08 | UP |
| GSU0978 | | 2.13804682 | 1.12E-05 | UP |
| GSU0979 | | 1.7317843 | 0.00165055 | UP |
| GSU0980 | | 1.76932259 | 0.00133175 | UP |
| GSU0981 | | 1.91428366 | 0.00013815 | UP |
| GSU0982 | | 2.07707704 | 9.71E-06 | UP |
| GSU0983 | | 1.93853319 | 1.25E-05 | UP |
| GSU0986 | | 2.24650469 | 1.70E-07 | UP |
| GSU0987 | | 2.53781159 | 1.11E-07 | UP |
| GSU0988 | | 2.30571888 | 1.09E-06 | UP |
| GSU0990 | | 3.50005658 | 1.13E-13 | UP |
| GSU0992 | | 3.01578802 | 8.60E-12 | UP |
| GSU1018 | | 2.31489175 | 8.26E-09 | UP |
| GSU1947 | | 1.59806831 | 0.00137395 | UP |
| GSU1948 | | 2.10062549 | 3.97E-06 | UP |
| GSU2750 | | 2.02389399 | 0.00049659 | UP |
| GSU2968 | | 3.59597344 | 3.34E-09 | UP |
| GSU3141 | | 1.84192789 | 1.21E-05 | UP |
| GSU3171 | | 3.72450908 | 1.94E-09 | UP |
| GSU3409 | | 2.92696187 | 6.34E-06 | UP |
| GSU3410 | | 2.06776312 | 0.00115908 | UP |
| GSU3489 | | 1.89866972 | 5.34E-06 | UP |
| GSU0071 | | -3.2185281 | 1.63E-12 | DOWN |
| GSU0081 | | -1.7206778 | 2.35E-05 | DOWN |
| GSU0216 | | -2.8770745 | 1.08E-10 | DOWN |
| GSU0545 | | -1.7528881 | 2.31E-05 | DOWN |
| GSU0919 | | -2.1246984 | 0.00011985 | DOWN |
| GSU1395 | | -2.2112398 | 2.23E-07 | DOWN |
| GSU1512 | | -2.3802995 | 2.01E-08 | DOWN |
| GSU2499 | | -1.9488119 | 4.43E-06 | DOWN |
| GSU2505 | | -4.978854 | 4.40E-32 | DOWN |
| GSU2585 | | -1.6994992 | 0.00010392 | DOWN |
| GSU2662 | | -4.201099 | 8.09E-19 | DOWN |
| GSU3085 | *yqfO* | -1.5288064 | 0.00012375 | DOWN |
| GSU3568 | *lnt-C* | -3.0603004 | 1.42E-12 | DOWN |
