# Supplementary material for: Global transcriptional analysis of Geobacter sulfurreducens gsu1771 mutant biofilm grown on two different support structures
Source: PLoS One. 2023 Oct 25;18(10):e0293359. doi: 10.1371/journal.pone.0293359 (PMC10599522; doi:10.1371/journal.pone.0293359)
Supplement: S1 Raw images — (PDF) [file pone.0293359.s005.pdf]

Global transcriptional analysis of *Geobacter sulfurreducens gsu1771* mutant biofilm grown on two different support structures

Juan B. Jaramillo-Rodríguez[1], Leticia Vega-Alvarado[2], Luis M. Rodríguez-Torres[1], Guillermo A. Huerta-Miranda[1], Alberto Hernández-Eligio[1,3*], Katy Juarez[1*]

[1]Departamento de Ingeniería Celular y Biocatálisis, Instituto de Biotecnología Universidad Nacional Autónoma de México, Cuernavaca, Morelos, México

[2]Instituto de Ciencias Aplicadas y Tecnología, Universidad Nacional Autónoma de México, Ciudad Universitaria, Ciudad de México, México

[3]Investigador por México, Consejo Nacional de Ciencia y Tecnología, Ciudad de México, México

[*]Corresponding author

E-mail: katy.juarez@ibt.unam.mx (KJ)

E-mail: alberto.hernandez@ibt.unam.mx (AH-E)

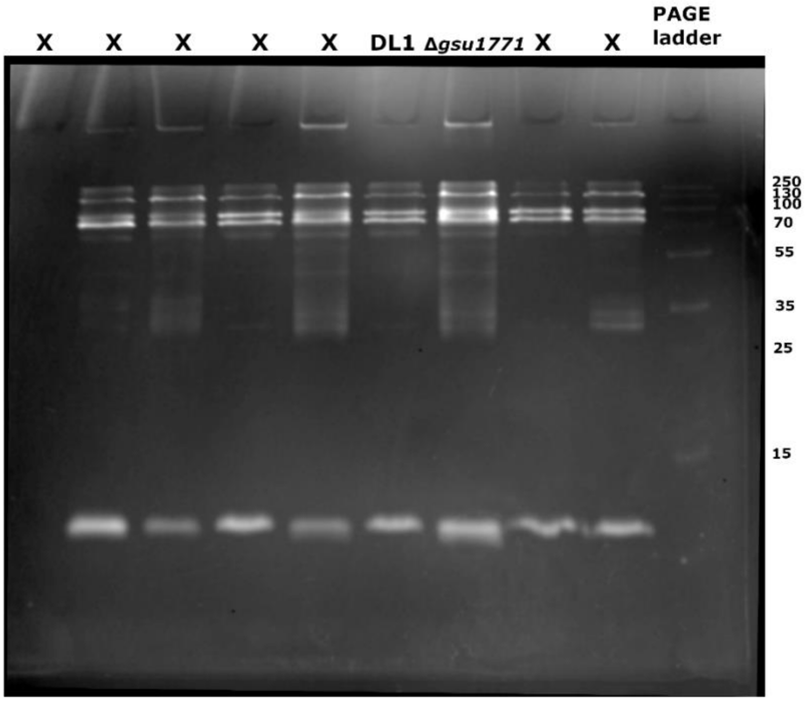

Fig 3A. Samples DL1, Δ*gsu1771.* Gel visualized using a Gel Doc DZ imager (Bio-Rad).

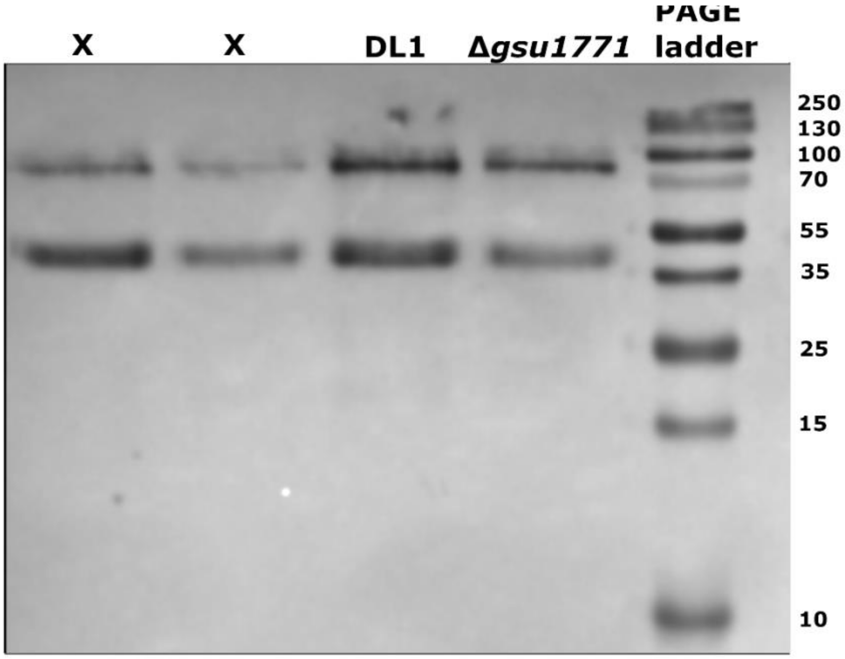

Fig 3B. Samples DL1, Δ*gsu1771.* OmcS immunoblot, nitrocellulose membrane visualized using a Gel Doc DZ imager (Bio-Rad).

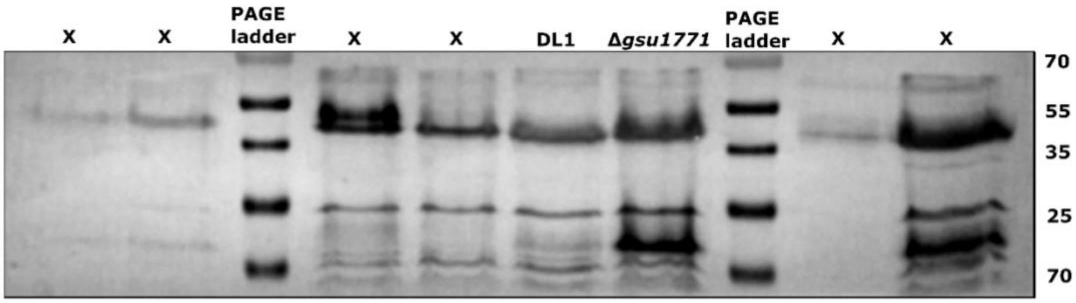

Fig 3C. Samples DL1, Δ*gsu1771.* OmcZ immunoblot, nitrocellulose membrane visualized using a Gel Doc DZ imager (Bio-Rad).

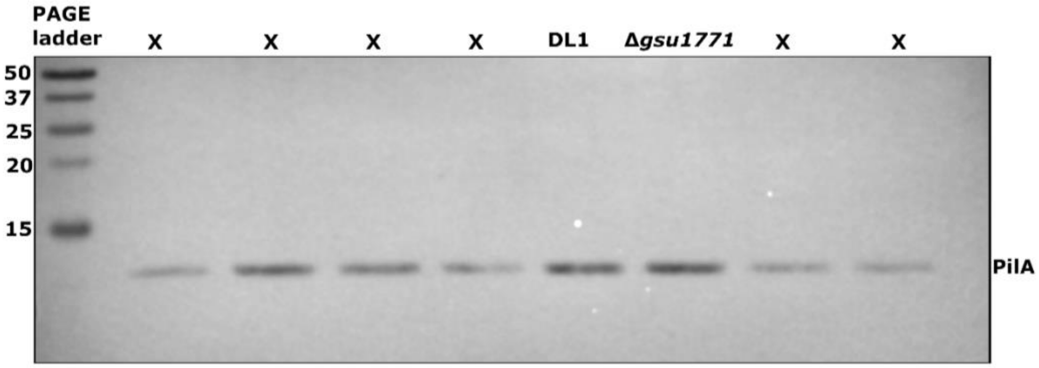

Fig 3D. Samples DL1, Δ*gsu1771.* PilA immunoblot, nitrocellulose membrane visualized using a Gel Doc DZ imager (Bio-Rad).

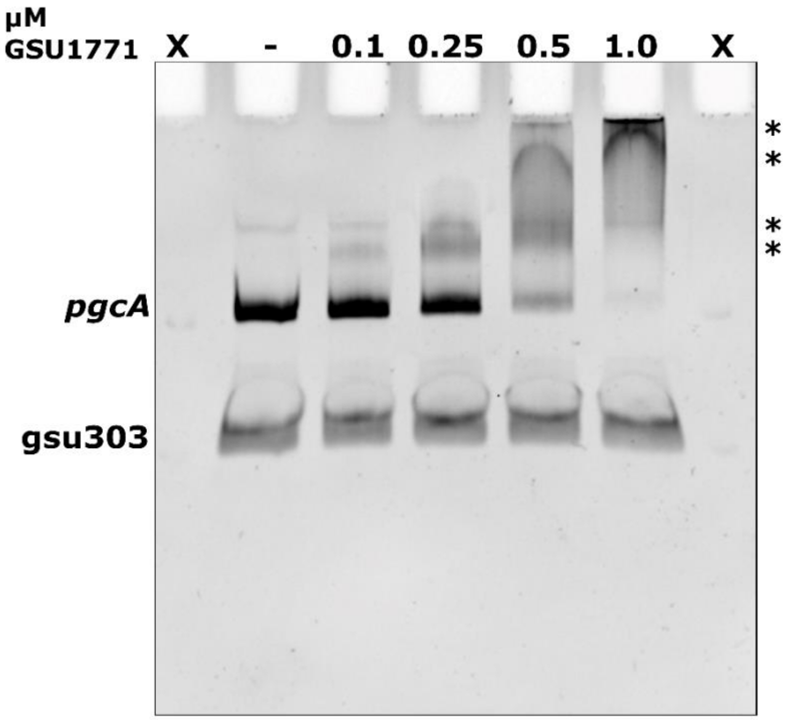

Fig 6. Gel from the EMSA experiment using the promoter region of *pgcA*. We used different concentrations of GSU1771 protein (0, 0.1, 0.25, 0.5, and 1 μM) gel visualized using a Gel Doc DZ imager (Bio-Rad).

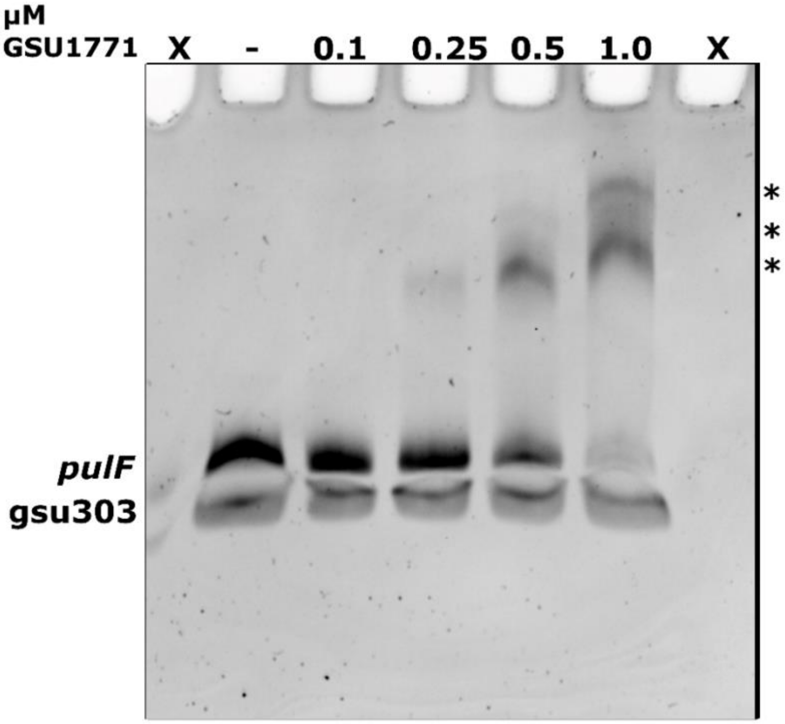

Fig 6. Gel from the EMSA experiment using the promoter region of *pulF*. We used different concentrations of GSU1771 protein (0, 0.1, 0.25, 0.5, and 1 μM) gel visualized using a Gel Doc DZ imager (Bio-Rad).

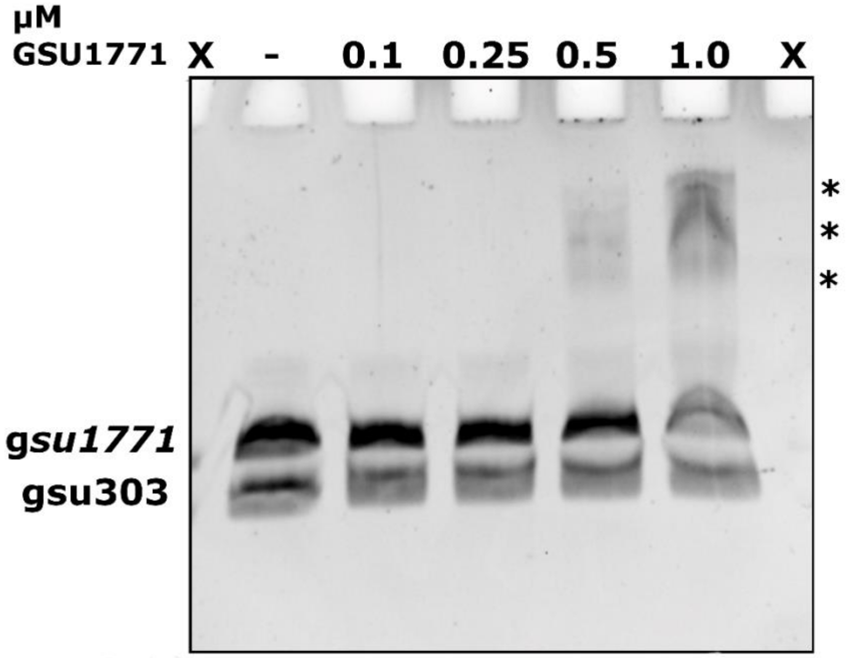

Fig 6. Gel from the EMSA experiment using the promoter region of *gsu1771.* We used different concentrations of GSU1771 protein (0, 0.1, 0.25, 0.5, and 1 μM) gel visualized using a Gel Doc DZ imager (Bio-Rad).

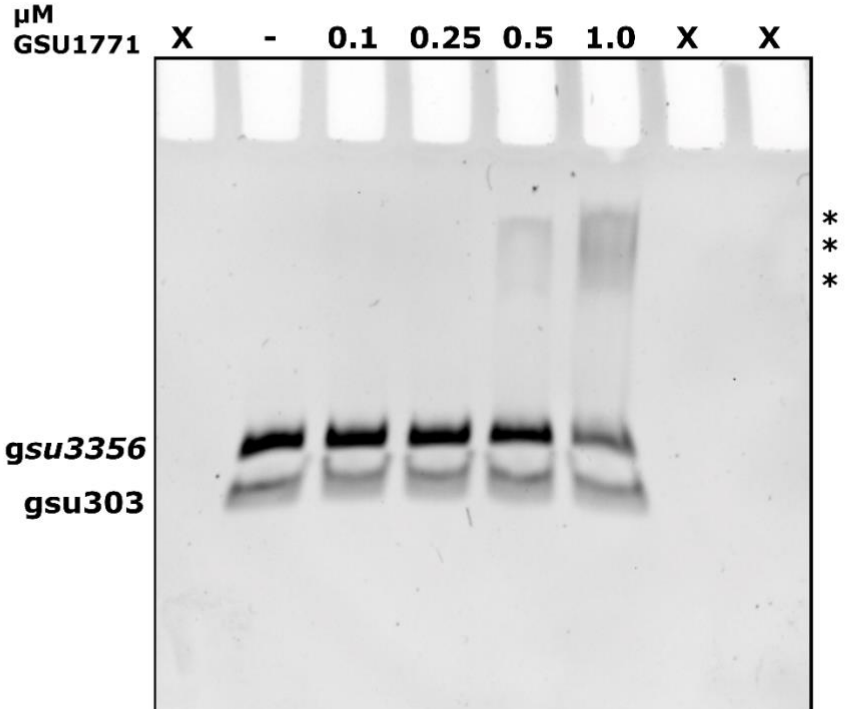

Fig 6. Gel from the EMSA experiment using the promoter region of *gsu3356.* We used different concentrations of GSU1771 protein (0, 0.1, 0.25, 0.5, and 1 μM) gel visualized using a Gel Doc DZ imager (Bio-Rad).

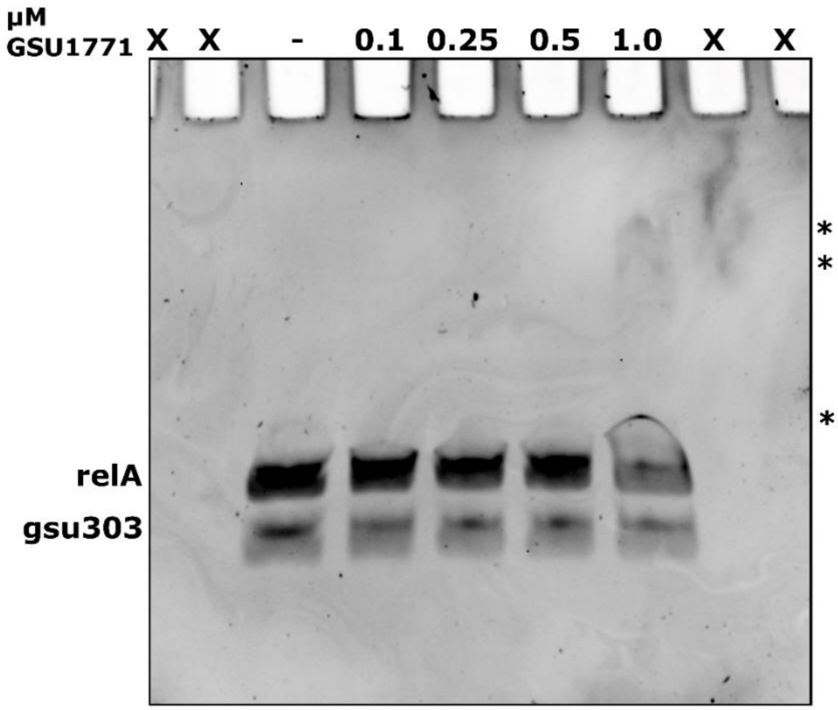

Fig 6. Gel from the EMSA experiment using the promoter region of *relA.* We used different concentrations of GSU1771 protein (0, 0.1, 0.25, 0.5, and 1 μM) gel visualized using a Gel Doc DZ imager (Bio-Rad).

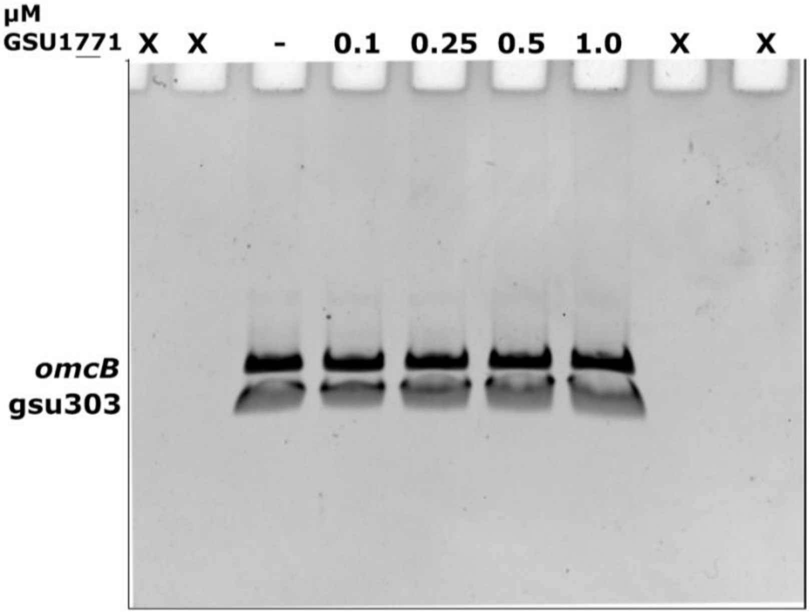

Fig 6. Gel from the EMSA experiment using the promoter region of EMSA *omcB.* We used different concentrations of GSU1771 protein (0, 0.1, 0.25, 0.5, and 1 μM) gel visualized using a Gel Doc DZ imager (Bio-Rad).

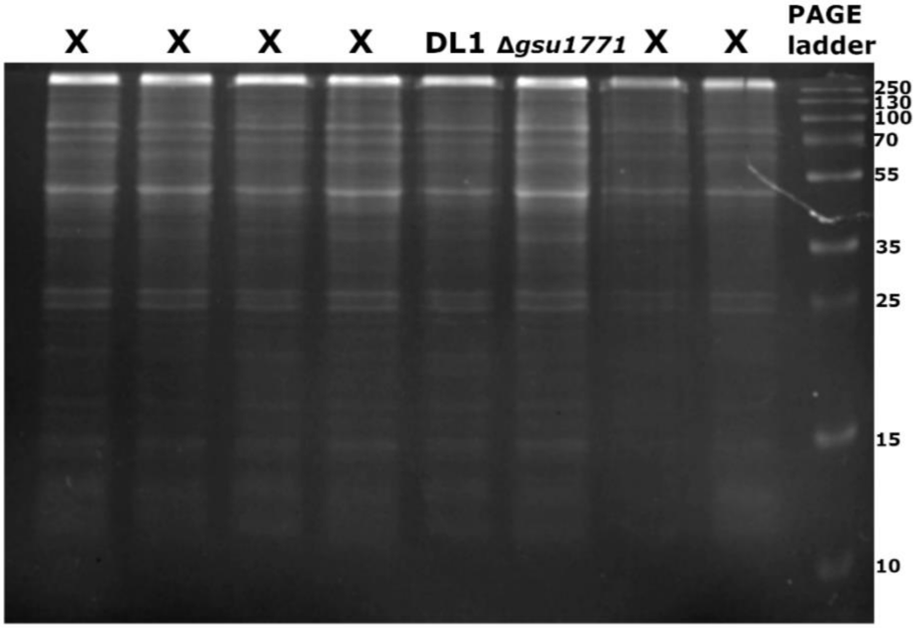

S1 Fig. A SDS PAGE of protein used as a loading control in heme-staining. Samples DL1 and Δ*gsu1771*. Gel staining with Coomassie blue and visualized using a Gel Doc DZ imager (Bio-Rad).

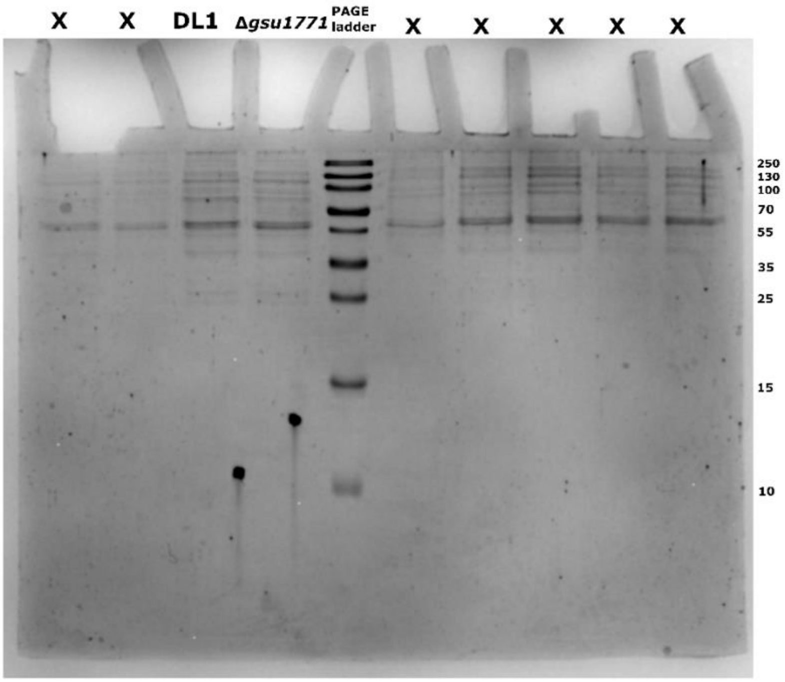

S1 Fig. B SDS-PAGE of protein used as a loading control in OmcS western blot. Samples DL1 and Δ*gsu1771.* Gel staining with Coomassie blue and visualized using a Gel Doc DZ imager (Bio-Rad).

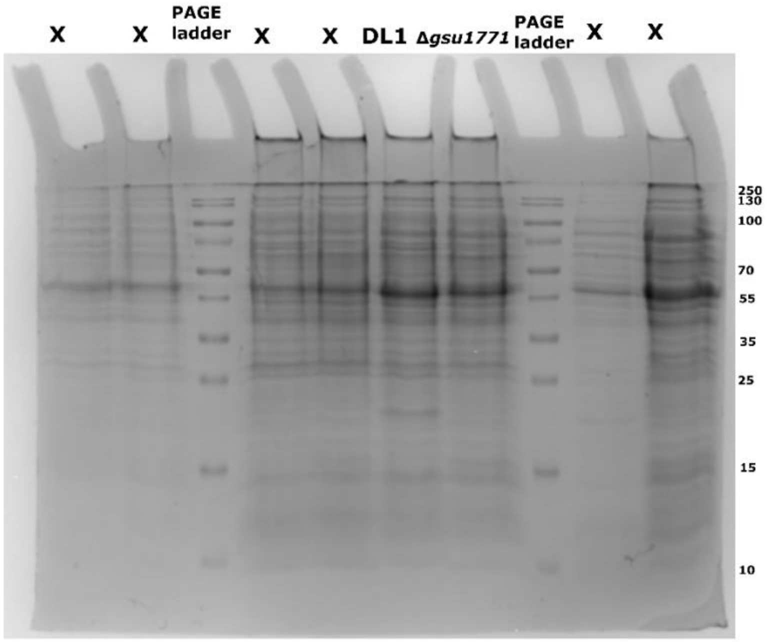

S1 Fig. C SDS-PAGE of protein used as a loading control in OmcZ western blot. Samples DL1 and Δ*gsu1771.* Gel staining with Coomassie blue and visualized using a Gel Doc DZ imager (Bio-Rad).

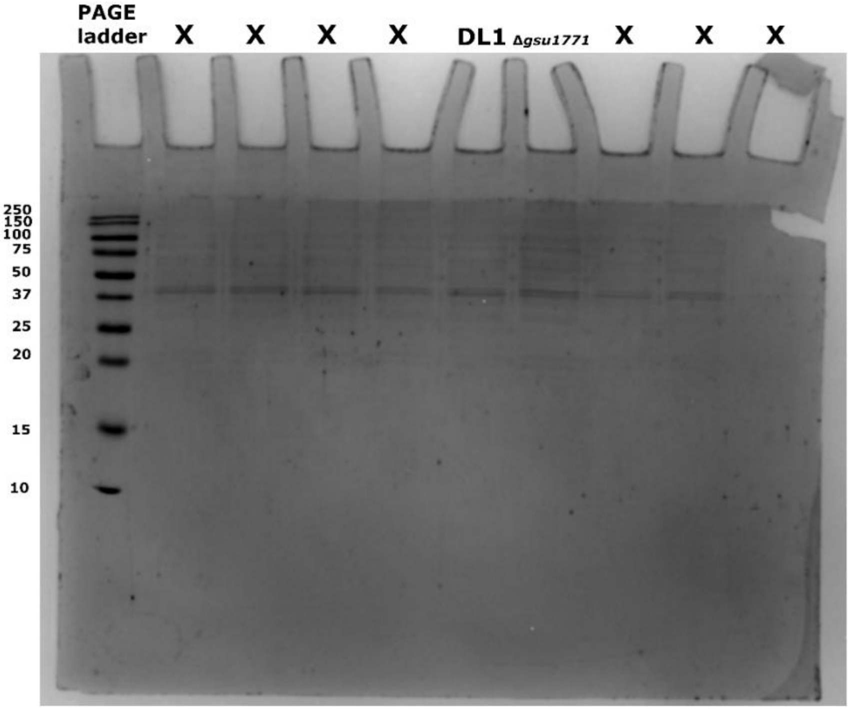

S1 Fig. D SDS-PAGE of protein used as a loading control in PilA western blot. Samples DL1 and Δ*gsu1771.* Gel staining with Coomassie blue and visualized using a Gel Doc DZ imager (Bio-Rad).
