# Supplementary material for: Global transcriptional analysis of Geobacter sulfurreducens gsu1771 mutant biofilm grown on two different support structures
Source: PLoS One. 2023 Oct 25;18(10):e0293359. doi: 10.1371/journal.pone.0293359 (PMC10599522; doi:10.1371/journal.pone.0293359)
Supplement: Attachment — Submitted filename: Response to Reviewers Jaramillo-Rodriguez et al.pdf [file pone.0293359.s006.pdf]

Journal Requirements:

When submitting your revision, we need you to address these additional requirements.
1. Please ensure that your manuscript meets PLOS ONE's style requirements, including those for file naming. The PLOS ONE style templates can be found at https://journals.plos.org/plosone/s/file?id=wjVg/PLOSOne_formatting_sample_main_body.pdf and https://journals.plos.org/plosone/s/file?id=ba62/PLOSOne_formatting_sample_title_authors_affiliations.pdf

> Response: Thanks, the manuscript has been edited according to PLOS ONE´s style requirements.

2. We note that the grant information you provided in the ‘Funding Information’ and ‘Financial Disclosure’ sections do not match.
When you resubmit, please ensure that you provide the correct grant numbers for the awards you received for your study in the ‘Funding Information’ section.

> Response: Thanks, the “Funding information” and “Financial Disclosure” has been corrected.

3. Please expand the acronym “PAPIIT-UNAM, CONACYT, PASPA-UNAM” (as indicated in your financial disclosure) so that it states the name of your funders in full.
This information should be included in your cover letter; we will change the online submission form on your behalf.

> Response: Thanks, the acronyms “PAPIIT-UNAM, CONACYT, PASPA-UNAM” were expanded and included in the cover letter.

4. Thank you for stating the following financial disclosure:
"KJ and AH-E received PAPIIT-UNAM (grant No IN212022). BJ-R received CONACYT-scholarship awarded during his master’s program GMH-M received CONACYT-postdoctoral fellowship (2322131). KJ received PASPA-UNAM for financial support during a sabbatical stay"
Please state what role the funders took in the study. If the funders had no role, please state: "The funders had no role in study design, data collection and analysis, decision to publish, or preparation of the manuscript." If this statement is not correct you must amend it as needed.
Please include this amended Role of Funder statement in your cover letter; we will change the online submission form on your behalf.

> Response: Thanks, the financial disclosure:
> "KJ and AH-E received PAPIIT-UNAM (grant No IN212022). BJ-R received CONACYT-scholarship awarded during his master’s program. GAH-M received CONACYT-postdoctoral fellowship (2322131). KJ received PASPA-UNAM for

financial support during a sabbatical stay" was eliminated from "Acknowledgment" in the manuscript, and we add the "Role of Funder" in the cover letter.

5. Thank you for stating the following in the Acknowledgments Section of your manuscript: "This study was financially supported by PAPIIT-UNAM (grant No IN212022). We thank Leticia Olvera, Veronica Jacinto, Ricardo Grande, Andres Saralegui, and Shirley Ainsworth for their technical support. BJ-R thanks CONACYT for the scholarship awarded during his master's program. GMH-M thanks CONACYT for the postdoctoral fellowship (2322131), KJ also thanks PASPA-UNAM for financial support during a sabbatical stay.."
We note that you have provided funding information that is not currently declared in your Funding Statement. However, funding information should not appear in the Acknowledgments section or other areas of your manuscript. We will only publish funding information present in the Funding Statement section of the online submission form.
Please remove any funding-related text from the manuscript and let us know how you would like to update your Funding Statement. Currently, your Funding Statement reads as follows: "KJ and AH-E received PAPIIT-UNAM (grant No IN212022). BJ-R received CONACYT-scholarship awarded during his master's program GMH-M received CONACYT-postdoctoral fellowship (2322131) KJ received PASPA-UNAM for financial support during a sabbatical stay". Please include your amended statements within your cover letter; we will change the online submission form on your behalf.

Response: Thanks, the funding information was removed from "Acknowledgments" Section in the manuscript, and the Funding Statement was added to cover letter to include this information in online submission.

6. Thank you for stating the following in your Competing Interests section: "NO authors have competing interests"
Please complete your Competing Interests on the online submission form to state any Competing Interests. If you have no competing interests, please state "The authors have declared that no competing interests exist.", as detailed online in our guide for authors at http://journals.plos.org/plosone/s/submit-now
This information should be included in your cover letter; we will change the online submission form on your behalf.

Response: Thanks, the "Competing Interests" were included in the cover letter for the correction in online submission.

7. In your Data Availability statement, you have not specified where the minimal data set underlying the results described in your manuscript can be found. PLOS defines a study's minimal data set as the underlying data used to reach the conclusions drawn in the manuscript and any additional data required to replicate the reported study findings in their entirety. All PLOS journals require that the minimal data set be made fully available. For more information about our data policy, please see http://journals.plos.org/plosone/s/data-availability.
Upon re-submitting your revised manuscript, please upload your study's minimal underlying data set as either Supporting Information files or to a stable, public repository and include the relevant URLs, DOIs, or accession numbers within your revised cover letter. For a list of

acceptable repositories, please see http://journals.plos.org/plosone/s/data-availability#loc-recommended-repositories. Any potentially identifying patient information must be fully anonymized.
Important: If there are ethical or legal restrictions to sharing your data publicly, please explain these restrictions in detail. Please see our guidelines for more information on what we consider unacceptable restrictions to publicly sharing data: http://journals.plos.org/plosone/s/data-availability#loc-unacceptable-data-access-restrictions. Note that it is not acceptable for the authors to be the sole named individuals responsible for ensuring data access.
We will update your Data Availability statement to reflect the information you provide in your cover letter.

Response: Thanks, the "Data Availability statement" was included in the cover letter for the correction in online submission.

8. PLOS ONE now requires that authors provide the original uncropped and unadjusted images underlying all blot or gel results reported in a submission's figures or Supporting Information files. This policy and the journal's other requirements for blot/gel reporting and figure preparation are described in detail at https://journals.plos.org/plosone/s/figures#loc-blot-and-gel-reporting-requirements and https://journals.plos.org/plosone/s/figures#loc-preparing-figures-from-image-files. When you submit your revised manuscript, please ensure that your figures adhere fully to these guidelines and provide the original underlying images for all blot or gel data reported in your submission. See the following link for instructions on providing the original image data: https://journals.plos.org/plosone/s/figures#loc-original-images-for-blots-and-gels.
In your cover letter, please note whether your blot/gel image data are in Supporting Information or posted at a public data repository, provide the repository URL if relevant, and provide specific details as to which raw blot/gel images, if any, are not available. Email us at plosone@plos.org if you have any questions.

Response: Thanks, the S1_raw_images.pdf archive with all original gels and blots was included in submission.

9. Please amend either the title on the online submission form (via Edit Submission) or the title in the manuscript so that they are identical.

Response: Thanks, the title of the manuscript and the online submission was revised and now is the same.

Reviewer's comments:

Reviewer's Responses point by point

Comments to the Author

1. Is the manuscript technically sound, and do the data support the conclusions?

The manuscript must describe a technically sound piece of scientific research with data that supports the conclusions. Experiments must have been conducted rigorously, with appropriate controls, replication, and sample sizes. The conclusions must be drawn appropriately based on the data presented.

Reviewer #1: Yes
Reviewer #2: Partly

2. Has the statistical analysis been performed appropriately and rigorously?

Reviewer #1: N/A
Reviewer #2: N/A

3. Have the authors made all data underlying the findings in their manuscript fully available?

The PLOS Data policy requires authors to make all data underlying the findings described in their manuscript fully available without restriction, with rare exception (please refer to the Data Availability Statement in the manuscript PDF file). The data should be provided as part of the manuscript or its supporting information or deposited to a public repository. For example, in addition to summary statistics, the data points behind means, medians and variance measures should be available. If there are restrictions on publicly sharing data—e.g., participant privacy or use of data from a third party—those must be specified.

Reviewer #1: Yes
Reviewer #2: No

4. Is the manuscript presented in an intelligible fashion and written in standard English?

PLOS ONE does not copyedit accepted manuscripts, so the language in submitted articles must be clear, correct, and unambiguous. Any typographical or grammatical errors should be corrected at revision, so please note any specific errors here.

Reviewer #1: Yes
Reviewer #2: Yes

5. Review Comments to the Author

Please use the space provided to explain your answers to the questions above. You may also include additional comments for the author, including concerns about dual publication, research ethics, or publication ethics. (Please upload your review as an attachment if it exceeds 20,000 characters)

Reviewer #1:

- The no of references should be reduced.

Response: We have already reduced the number of them; however, the large number is due to the description of the genes differentially expressed, some with function studied in other bacteria.

The manuscript contains voluminous data that often confuses the reader as the objectives of the work are not mentioned clearly in the Introduction. However, the work is good and deserves publication after minor revision.

Response: We add the aim of this work in the introduction.

Reviewer #2:

Appears to be an interesting paper following up on prior observations. The entire paper is based on transcriptional analysis, but the reviewer is unable to review it without access to RNAseq data. NCBI DEO database data is locked until 2025 without the secure token.

Response: Thanks, the data generated from the transcriptome analysis uploaded to the Gene Expression Omnibus repository has been released for inquiries and download. NCBI Gene Expression Omnibus database under accession number GSE223184.

No data on depth, % that was rRNA, or quality of RNA read data.

Response: Here are the data requested summarized in a table.

Quality Report

Sample	Total reads	Mapped reads	% Mapped reads	Coverage	Ribosomal	% Ribosomal	Total of reads for mapped	Phred quality score
GSU1771_Electrode_rep1	33924210	32721516	96.45%	667.0766555	29875867	88.07%	2845649	> 30
GSU1771_Electrode_rep2	34832138	33682323	96.70%	684.9299106	31299847	89.86%	2382476	> 31
WT_Electrode_rep1	33731692	32546760	96.49%	663.2910327	30551080	90.57%	1995680	> 32
WT_Electrode_rep2	34440070	33063486	96.00%	677.2203896	31032198	90.10%	2031288	> 33
WT_Glass_rep1	23031764	21614730	93.85%	452.8904903	20110224	87.32%	1504506	> 34
WT_Glass_rep2	23417196	22195529	94.78%	460.4695228	20817113	88.90%	1378416	> 35
GSU1771_Glass_rep1	21392342	19837513	92.73%	420.6533315	16855883	78.79%	2981630	> 36
GSU1771_Glass_rep2	24268702	23084357	95.12%	477.2133106	20055882	82.64%	3028475	> 37

Also, unable to accept without a supplementary table of --entire-- transcriptional data. Only selected data is provided about author-selected genes. This selection is based on a "Custom script" used for analysis, unable to verify or repeat analysis. Unable to use data for future work if only subset chosen by unrepeatable analysis.

Response: The data of all the differentially expressed genes detected in the RNAseq analysis are reported in Supplementary Tables 2 and 3.

Please check Fig 5 -- if only a few microamps of current were produced from such large 65 cm^2 electrodes, the biofilms are barely alive and should not be used. It is very odd that the person who performed crucial experiments needed to produce the key data in this paper is not an author or part of any part of the analysis. If there is no author in this paper who understands the difference between good and bad growth on the electrode, it is suspect.

Response: Thanks for the observation. KJ carried out the MFC experiments and Trevor L. Woodard supervised the work. Biofilms with viable cells were scraped (photos of the graphite electrode are attached).

The low current data obtained may be due the application of a potential of 60 mV instead of 300 mV (normally used in these experiments). This decision was made because at the same time experiments were being carried out with FTO electrodes and we set the same conditions in order to compare both supports. In previous studies in our working group, we found that at 60 mV *G. sulfurreducens* biofilms formed on FTO electrodes reached limiting current in turn-over conditions in Cyclic Voltammetry experiments (Hernández-Eligio et al., 2022). We believe that this is the reason for the difference. As can be seen with respect to the WT strain there is an increase of 20%. On the other hand, these biofilms were collected and used for RNA extraction and RNA seq experiments (All quality data are adequate and indirectly reflect that the biofilms were active).

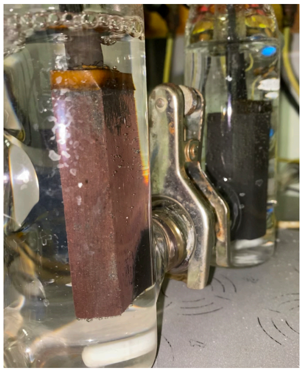
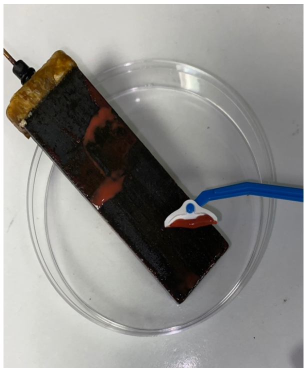

6. PLOS authors have the option to publish the peer review history of their article (what does this mean?). If published, this will include your full peer review and any attached files.
If you choose “no”, your identity will remain anonymous but your review may still be made public.
Do you want your identity to be public for this peer review? For information about this choice, including consent withdrawal, please see our Privacy Policy.
Reviewer #1: No
Reviewer #2: No
